# Effect of TiO_2_ and CaO Addition on the Crystallization and Flexural Strength of Novel Leucite Glass-Ceramics

**DOI:** 10.3390/ma17143422

**Published:** 2024-07-11

**Authors:** Jamila Almuhamadi, Mustafa H. Almusali, Xiaohui Chen, Antonios L. Theocharopoulos, Hawraa F. Alostath, Natalia Karpukhina, Michael J. Cattell

**Affiliations:** 1 Centre for Oral Bioengineering, Faculty of Medicine and Dentistry, Queen Mary University of London, Turner Street, London E1 2AD, UK; j.almuhamadi@zu.edu.ly (J.A.); m.h.e.almusali@qmul.ac.uk (M.H.A.); h.alostath@qmul.ac.uk (H.F.A.); n.karpukhina@qmul.ac.uk (N.K.); 2 Division of Dentistry, School of Medical Sciences, The University of Manchester, Manchester M13 9PL, UK; xiaohui.chen@manchester.ac.uk; 3 Biomedical Sciences Department, Dental Technology Division, University of West Attica, Agiou Spyridonos Street, 122 43 Athens, Greece; antheoch@uniwa.gr

**Keywords:** crystallization, flexural strength, glass-ceramics, leucite, nucleation

## Abstract

The aim of this study was to investigate the effects of TiO_2_/CaO addition on the crystallization and flexural strength of leucite glass-ceramics (GC). Synthesis of translucent and high strength GCs is important for the development of aesthetic and durable dental restorations. To achieve this, experimental aluminosilicate glasses (1–3 mol% TiO_2_ and CaO (B1, B2, B3)) were melted in a furnace to produce glasses. Glasses were ball milled, screened and heat treated via crystallization heat treatments, and characterized using XRD, differential scanning calorimetry, dilatometry, SEM and biaxial flexural strength (BFS). Increasing nucleation hold time (1–3 h) led to a reduction in crystallite number for B2 and B3 GC, and significant differences in leucite crystal size at differing nucleation holds within and across test groups (*p* < 0.05). A high area fraction of leucite crystals (55.1–60.8%) was found in the GC, with no matrix microcracking. Changes in the crystal morphology were found with higher TiO_2_/CaO addition. Mean BFS of the GC were 211.2–234.8 MPa, with significantly higher Weibull modulus (m = 18.9) for B3 GC. Novel glass compositions enriched with TiO_2_/CaO led to crystallization of leucite GC of high aspect ratio, with high BFS and reliability. The study’s findings suggest a potential high performance translucent leucite GC for use in the construction of dental restorations.

## 1. Introduction

Leucite (KAlSi_2_O_6_) was first introduced in dental porcelain to control its thermal expansion coefficient, making it thermally compatible with metals [1]. Leucite crystals dispersed in a thermally compatible glass also have applications as a reinforcing agent in all-ceramic restorations [2] and can be synthesized by the incongruent melting of potash feldspar or synthetically produced as a powder [3], utilizing various methods such as sol-gel [4] or hydrothermal synthesis [5,6]. Leucite incorporation into a base glass was initially achieved by blending low and high expansion powdered feldspar frits [1], or later by dispersion [7]. This incorporation results in a dispersion strengthening/toughening effect [8,9] due to the crack-pinning and diverting ability [10] of the leucite crystals and related to their thermal relationship with the residual glass and crystal size [11,12]. Leucite crystals can also be grown in an appropriately designed aluminosilicate glass by utilizing controlled heat treatments [13,14,15]. This allows careful control over the microstructure and enhancement of properties, including increased flexural strength, [15,16] reduced wear [17] and translucency [18]. Glass-ceramics with these properties are extremely desirable for the construction of dental restorations (inlays/onlays, crowns and bridges) to prevent fracture, enamel tooth wear and to recreate the translucency, texture, and color of natural teeth [18].

The controlled crystallization of leucite can be catalyzed by introducing specific nucleating agents (TiO_2_, ZrO_2_) [19] into a glass. These agents promote very fine-scale phase separation, and crystallization can be induced within or from the surface of these phase separated domains [20]. They can also act as a sub-microscopic catalyst for heterogeneous nucleation and growth of the major crystalline phase [12,21]. There is also the potential of epitaxial growth in aluminosilicate glass systems on Zr_(1−x)_Ti_(1+x)_O_4_ crystals [22]. 

Titanium dioxide (2–20 wt.%) [23] is an efficacious nucleation agent that is effective in a variety of glass compositions. Increased TiO_2_ content (>20 wt.%), however, causes TiO_2_ crystals to precipitate in the glass matrix, causing decreased nucleation and growth of the crystalline phase [24]. It is therefore important to understand and control the optimum TiO_2_ content in a glass for efficient and controlled crystallization for the required application. The authors have previously studied an aluminosilicate glass with 1.3 mol% TiO_2_ content, reporting crystallization of leucite via phase separation and the growth of leucite on Na/Ca titanates for the first time [25]. The presence of TiO_2_ in conjunction with calcium oxide (CaO) appears to have a catalytic effect on crystallization, favoring the formation of a titanite phase (CaTiSiO_5_) [26,27]. It is advantageous to examine the synergistic effect between these cations in an aluminosilicate glass more systematically, to understand any beneficial crystallization effects that may be gained in this important category of materials.

The aim of this study is therefore to synthesize a series of aluminosilicate glasses with increases in TiO_2_ and CaO content, and to assess their influence on the crystallization and flexural strength of these restorative materials to evaluate their potential for dental applications. 

## 2. Materials and Methods

### 2.1. Glass Synthesis

The glass compositions (Table 1) were designed using Appen factors [28] with general Formula (1).

X = Σi αi Pi(1)

where X indicates the thermal expansion coefficient or factor of interest of the glass, αi is the characteristic Appen factor and Pi is the mole percent concentration of each oxide. The fusion temperature contribution of each oxide was also considered to estimate glass processing temperatures [29]. 

Original glasses were designed to ensure a matched thermal expansion and refractive index with the leucite phase [30], and to ensure processing temperatures were suitable for the synthesis of dental restorations.

Titanium dioxide and calcium oxide were introduced into aluminosilicate glasses at 1–3 mol%. High purity reagents (Table S1) were weighed with a digital microbalance (Explorer, OHAUS, Greifensee, Switzerland) to fabricate glass batches for B1, B2 and B3 glasses (Table 1).

The batches were mixed via ball milling for two hours with alumina grinding media (26.4 mm diameter). The mixed batches were then sequentially transferred to a platinum/rhodium (90/10) crucible, heated in an electrical furnace (UAF 16/10, Lenton, Hope Valley, UK) at a rate of 10 °C/min to 1550 °C, held for 5 h and air quenched (1 min). The glasses were then placed in a preheated furnace (Tris Burnout Furnace, Dentalfarm, Italy) and annealed at 500 °C for 1 h before cooling to room temperature. The glasses were again crushed and ball milled for 2 h with 26.4 mm diameter alumina grinding media. The glass powders were re-homogenized by being melted again in the platinum/rhodium crucible at a rate of 10 °C/min to 1550 °C, were held for 2 h, air quenched, annealed for 2 h at 500 °C, and subsequently cooled to room temperature in a furnace. Glasses were crushed and ball milled for 2 h with a mixture of 19.0 mm and 26.4 diameter alumina grinding media. The glass powders were screened on a sieve shaker (Retsch, VS1000, Haan, Germany) (1 h, 60 Hz) through 300 µm, 125 µm and 45 µm sieves (Endecotts Ltd., London, UK).

### 2.2. Differential Scanning Calorimetry

Differential Scanning Calorimetry (DSC) was carried out using a Stanton Redcroft DSC 1500 (Rheometric Scientific, Epsom, UK). The instrument was calibrated for a 20–1300 °C temperature range. Weighed glass powder samples (0.05 g) were placed into a platinum crucible on the DSC stage next to a matched platinum crucible with alumina powder (0.05 g). Glass powders previously sieved through 300, 125 and 45 μm were ramped from 25 °C to 1200 °C at 20 °C/min under a nitrogen atmosphere. All DSC results were processed using the dedicated Infinity PRO Software (ver. 4.2.140, Instrument Specialists Inc., Twin Lakes, WI, USA) to enable peak crystallization temperature (Tp) determination.

To evaluate the crystallization kinetics of the glasses and the activation energies of crystallization, powder samples (125 μm) were ramped from 25 °C to 1200 °C at heating rates of 5, 10, 20 and 30 °C/min. The Kissinger Equation (2) was used [31]:

In (φ/Tp2) = −E/RTp + constant.(2)

where φ is the heating rate, R is the gas constant; Tp is the crystallization peak temperature and E is the activation energy of crystallization. The activation energy is obtained from the slope of the plot of In (Tp2/φ) versus 1000/Tp.

### 2.3. Crystallisation Studies

The glass powders (125 μm) were prepared as compacts by mixing 3 g powder with 0.5 mL modelling liquid (VITA, C.H.B 24066, Vita Zahnfabrik, Bad Sackingen, Germany) into slurry. The slurry was transferred to a steel mold (27 × 6 mm cross section) with a plunger, where it was condensed by manual vibration and tissue-dried for 30 s to remove excess moisture. The slurry was then pressed in a hydraulic press (Quayle Dental, Worthing, UK) for 1 min at 3 × 10^5^ Pa pressure. The powder compacts were removed from the mold, sequentially placed on platinum foils and inserted into a furnace (25 °C) (Lenton UAF16/10, Hope Valley, UK). The compacts were subjected to two-step (nucleation and growth) heat treatments (Table 2) and then air quenched. A temperature of 30 °C above the dilatometry determined Tg (Section 2.8) was selected as the nucleation temperature. Differential Scanning Calorimetry (DSC) determined Tps for glass powders (125 μm), which were used as the crystal growth temperatures for each of the glasses. To assess the effect of different nucleation holds, all glass specimens were ramped at 20 °C/min and held at the nucleation temperatures for 0.5, 1, 2 and 3 h, followed by 1 h of holding at the crystal growth temperature before quenching in air.

### 2.4. Glass-Ceramic Powder Production for Flexural Strength Specimen Fabrication

Glass powders (125 μm) produced in Section 2.1 were placed on custom-made investment trays (IPS Press Vest investment material, Ivoclar-Vivadent, Schaan, Liechtenstein) and heat treated (UAF 16/10, Lenton, Hope Valley, UK) using two-step heat treatment temperatures according to Table 2. Both the nucleation and crystal growth holds were set to 1 h. These optimal heat treatments were based on the results of the crystallization studies and quantitative image analysis data from SEM. At the end of the heat treatments, the trays were quenched in air and left to cool to room temperature. The glass-ceramics were surface-cleaned, crushed and then ground to powders in a ball mill for 2 h (Pascal Engineering Ltd., London, UK) using a mix of 19.0 mm and 26.4 mm alumina grinding media. The glass-ceramic powders were then screened through a 125 μm sieve (Endecotts Ltd., London, UK).

### 2.5. X-ray Diffraction

Glass and glass-ceramic powders were analyzed using the X’Pert Pro X-ray diffractometer (Panalytical, B.V., Almelo, The Netherlands). Bragg-Brentano flat plate geometry and Cu Kα radiation (λ1 = 1.54059 Å and λ = 1.54442 Å) was used. Data were collected from 5 to 120 degrees (2θ), with the X’ Celerator in continuous mode, giving data equivalent to a count time of 200 s and an interval of 0.0334° 2θ on a normal diffractometer. Phase analysis was carried out using the PANalytical X’Pert high score plus software (version 1.0, Philips Analytical, Almelo, The Netherlands) (ICDD: PDF-4 database). The structural model of tetragonal leucite (ICDD: 00-038-1423) was used for phase identification.

Crystal strain calculations were calculated using the XRD data and Equation (3) below was used [32].

(3) $$\varepsilon_{a}=\frac{a-a_{0}}{a_{0}}\;\;\;\;\;\;\;\;\;\;\;\varepsilon_{c}=\frac{c-c_{0}}{c_{0}}$$

$\varepsilon_{a}$ and $\varepsilon_{c}$ is the strain of leucite in the a– and c–axes, where the mean unit cell dimensions of tetragonal leucite (for glass-ceramic B1, B2, B3) was a and c, and the unit cell dimensions of reference tetragonal leucite (ICDD: 00-038-1423) were a_0_ and c_0_.

### 2.6. Flexural Strength Specimen Fabrication

Thirty glass-ceramic disc specimens were fabricated per test group (B1, B2 and B3). Glass-ceramic powder (1 g) was weighed using a microbalance (Metter PC 180, Greifensee, Switzerland), mixed with 3 mL of liquid (Vita modelling liquid, Vita, Bad Sackingen, Germany), placed in a special cylindrical hollow steel mold with a plunger (16 mm diameter), condensed with a tissue for 30 s and then placed in a dental hydraulic press (Silfradent, Santa Sofia, Italy) under 1 × 10^5^ Pa pressure for 1 min. The samples were placed in a pre-heated (538 °C) porcelain furnace (Multimatt MCII, Dentsply, Konstanz, Germany) and sintered at a rate of 38 °C/min to 1100 °C (B1, B2) or 1110 °C (B3) for 2 min under vacuum. The specimens were wet lapped to a thickness of 2 mm on an automatic lapping machine (Knuth-Rotor-3, Struers, Ballerup, Denmark) using P320, P600, P800 and P1000 grit SiC papers.

### 2.7. Biaxial Flexural Strength Testing

Biaxial flexural strength testing was carried out using the ball-on-ring test. Glass-ceramic disc specimens were centrally loaded onto a 10 mm diameter knife-edge support via a 4 mm diameter spherical ball indenter at a crosshead speed of 1mm/min until fracture, in an Instron testing machine (5567/h1580, Instron, Buckinghamshire, UK). A thin plastic sheet (0.03 mm) had been placed between the loading indenter and the specimen to evenly distribute the load. The BFS was calculated using the Timoshenko and Woinowsky-Krieger equation [33].

(4) $$\sigma_{max}=\frac{P}{h^{2}}\left\{\left(1+v\right)\left[0.485\times In\left(\frac{a}{h}\right)+0.52\right]+0.48\right\}\;$$

where: σ_max_ = maximum tensile stress, P = load at fracture, h = thickness of the specimen, a = the radius of knife-edge support and v = Poisson’s ratio of 0.25.

Thirty heat-pressed IPS Empress Esthetic specimens previously tested [16] using the same method were used as a commercial leucite glass-ceramic comparison group. To compare glass-ceramic test groups, a one-way ANOVA and Tukey’s multiple-comparison test was used (*p* < 0.05) (Sigma Stat ver. 2.03, SPSS Inc., Chicago, IL, USA). Characteristic strength and Weibull m values (WinSMITH™ Weibull and Visual 2.0M, Fulton Findings™, Torrance, CA, USA) were compared for the overlap of their double-sided confidence intervals at the 95% level to determine differences between test groups.

### 2.8. Dilatometry

Glass frit was cut into blocks (6 mm × 6 mm × 25 mm) using a plate saw (Struers Acutom 2, Struers Ltd., Glasgow, Scotland, UK). Glass-ceramic specimens were made by compacting glass-ceramic powder and sintering in a porcelain furnace. Specimens were finished with silicon carbide grinding papers (P320 and P600). The thermal expansion coefficient (TEC) and the glass transition temperature (Tg) of the specimens were measured using a differential dilatometer (DIL 402PC, Netzsch Instrument, Selb, Germany) at a heating rate of 3 °C/min within the temperature range of 25–1200 °C, and softening point protection was applied during measurement. The thermal expansion was calculated using the general Formula (5):

α = (ΔL/L_o_)/ΔT (5)

where ΔL is the change in length, L_o_ is the original length and ΔT is the change in temperature.

### 2.9. Secondary Electron Imaging

Polished and etched (0.1% hydrofluoric acid, 60 s) glass-ceramic specimens from the crystallization studies (Section 2.3) were gold-coated (Agar Auto Sputter Coater, Agar scientific Ltd., Stansted, UK) and viewed under secondary electron imaging (10 kV accelerating voltage, 10 mm working distance) with a field emission scanning electron microscope (FEI Inspect F, Oxford Instruments, Oxfordshire, UK). Photomicrographs were scanned using a pen tablet (CTL-460, Wacom Co., Ltd., Beijing, China) in combination with image analysis software (Sigma Scan Pro 5.0, Systat Software, Inc., Chicago, IL, USA) to measure the leucite crystal size and area fraction (total area = 3080.9 µm^2^). A one-way ANOVA on ranks and Kruskal-Wallis multiple comparison tests (*p* < 0.05) (SPSS ver. 29.0.1.0 (171), IBM Corp, Armonk, NY, USA) were carried out on the median crystal area size data to compare the leucite crystal sizes after 0.5, 1, 2, and 3 h nucleation hold times (and a 1 h crystallization hold) within and between glass-ceramic test groups (*p* < 0.05).

## 3. Results

### 3.1. DSC Results

The peak crystallization temperatures (Tp) determined for glasses B1–B3 (powder size = 125 μm) were: Glass B1: 868 °C, Glass B2: 903 °C and Glass B3: 916 °C. There was a shift in Tp towards higher temperatures with increasing mol% of TiO_2_/CaO addition. DSC plots run for differing particle sizes (Supplementary Materials, Figures S2–S4), show peak shifts indicating surface crystallization mechanisms. The magnitude of these Tp shifts for B3 (PS = 45 and 300 μm) were more limited (1 °C difference).

**Figure 1:**
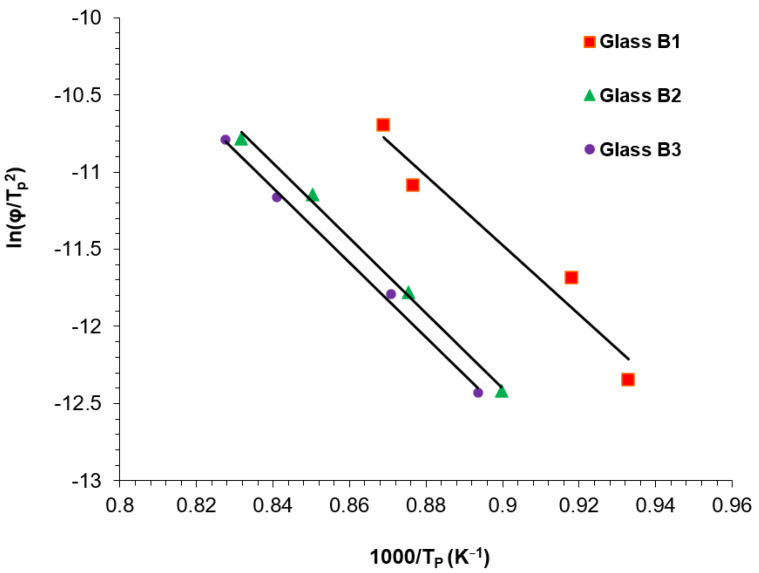
Kissinger plots for the experimental glasses.

The Kissinger plots for the activation energy determination are shown in Figure 1. The activation energies calculated were: B1 (186.8 KJ·mol^−1^, r^2^: 0.948), B2 (201.4 KJ·mol^−1^, r^2^: 0.996) and B3 (201.8 KJ·mol^−1^, r^2^: 0.997). There was an increase in the calculated activation energy with increasing addition of TiO_2_/CaO mol% in the glasses.

### 3.2. Dilatometry Results

The results of the dilatometry tests for the glass and glass-ceramics are shown in Table 3. There was an increase in Tg with increasing mol% of TiO_2_/CaO for both glass and glass-ceramics. Glass predictions for TEC agreed with the measured values (0.31–0.39 × 10^−6^/K differences, Table 3).

### 3.3. XRD Results

**Figure 2:**
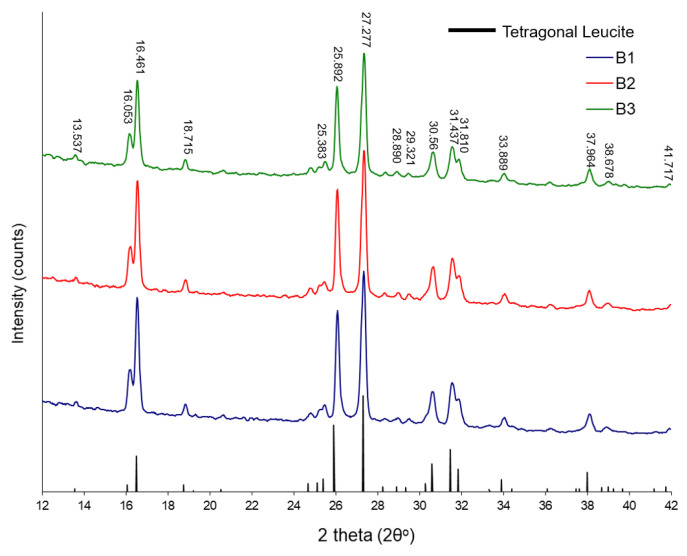
XRD plots for the experimental glass-ceramics.

All starting glasses were characterized as amorphous (Supplementary Materials, Figure S1). The XRD indicates a bulk tetragonal leucite phase for the glass-ceramics (1 h nucleation and growth holds, Figure 2) and changes in their unit cell dimensions (Table 4). Details of the unit cell dimension calculations can be seen in Table S2.

### 3.4. Scanning Electron Microscopy Results

**Figure 3:**
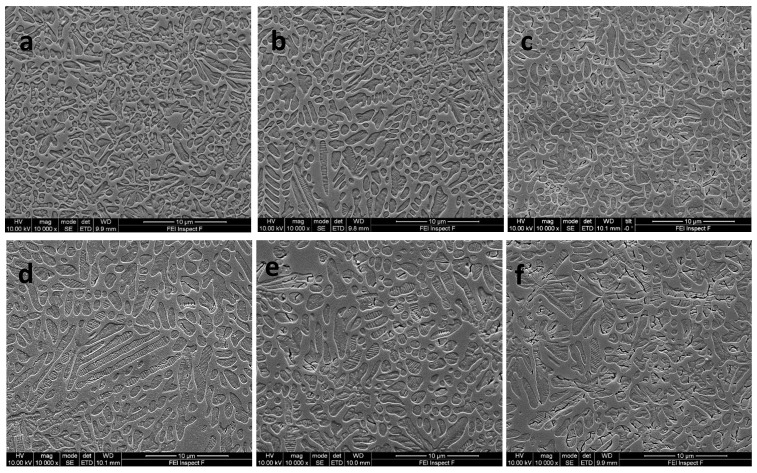
SEM photomicrographs of the glass-ceramics (GC) at different nucleation holds: (**a**) GC-B1 at 0.5 h hold showing mixed morphology of spherical crystals and crystal ripening; (**b**) GC-B1 at 3 h hold showing a mixture of elongated and spherical leucite crystals; (**c**) GC-B2 at 0.5 h hold showing a mixture of spherical leucite crystals and coalescence with signs of crystal microcracking; (**d**) GC-B2 at 3 h hold showing a mixture of elongated and spherical leucite crystals; (**e**) GC-B3 showing spherical and elongated crystals in the glassy matrix; (**f**) GC-B3 at 3 h hold showing leucite crystal ripening and cross-shaped crystal structures.

**Figure 4:**
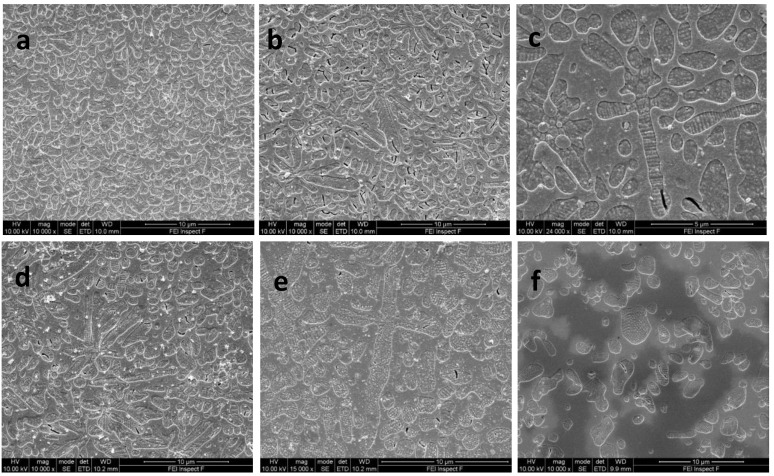
SEM photomicrographs of leucite glass-ceramics after 1 h nucleation holds/1 h crystal growth hold showing: (**a**) GC-B1 showing a dense dispersal of spherical leucite crystals; (**b**) GC-B2 indicting spherical and elongated leucite crystals and (**c**) sparse cross-shaped crystals; (**d**) GC-B3 showing a mixture of spherical and elongated leucite crystals and (**e**) cross-shaped crystals; (**f**) IPS Empress Esthetic glass-ceramic with spherical-shaped leucite crystals.

The results of the SEM and quantitative image analysis on different nucleation holds (0.5–3 h), followed by 1 h growth holds (Table 2), are shown in Figure 3a–f and Figure 4a–f, with statistical differences in crystal size within and across groups in Table 5. B1 glass-ceramics (GC) produced the highest crystal number and smallest median crystal sizes after all nucleation holds, compared with the other test groups (*p* < 0.05). There was a statistically significant (*p* < 0.05) increase in median crystallite size between B1 and B2, and between B1 and B3 GC, at all nucleation hold times (Table 5, Figure 3a–d). The longer nucleation hold time (3 h) for B3 GC encouraged crystallization of elongated and cross-shaped structures, and a significant increase in median crystal size (*p* < 0.05) and reduction in crystal number compared with all GCs evaluated at different hold times (Figure 3f, Table 5).

SEM photomicrographs of B1–B3 GCs at 1 h nucleation holds, followed by 1 h growth holds and IPS Empress Esthetic GC, are shown in Figure 4a–f. Glass-ceramic B1 showed a dense dispersal of spherical leucite crystals (Figure 4a). Glass-ceramics B2 (Figure 4b,c) and B3 (Figure 4d,e) showed a mix of spherical and elongated leucite crystals along with cross-shaped crystal structures. IPS Empress Esthetic exhibited a lower area fraction of spherical and irregularly shaped leucite crystals (Figure 4f).

### 3.5. BFS Results

The results of the BFS are shown in Table 6. All experimental glass-ceramics had significantly (*p* < 0.05) higher mean biaxial flexural strengths and characteristic strengths compared with the commercial comparison group (IPS Empress Esthetic). Significant differences (*p* < 0.05) between test groups can be seen in Table 6. Glass-ceramic B3 had the highest Weibull modulus (*p* < 0.05). Glass-ceramics B1 and B3 did not have significantly different Weibull moduli (*p* > 0.05), however B1 Weibull modulus was higher than the commercial comparison group (*p* < 0.05), while B2 Weibull modulus was not (*p* > 0.05).

## 4. Discussion

Glass formulations were synthesized in this work using Appen factors to predict the glass TEC, indicating a similar trend between predicted and measured values (Table 3), making this a useful tool to design glass properties prior to crystallization. Previous work [25] indicated that an aluminosilicate glass containing CaO (1.8%), TiO_2_ (1.3%) and Na_2_O (2.4%) induced phase separation and the growth of leucite on Na/Ca titanate seeds (20–50 nm). This produced a high-volume fraction (65.5–69.3%) of fine (mean crystal size = 0.85–0.599 μm^2^) spherical crystallites. There were signs of spherical inclusions within, and associated with, the crystallites in the current formulations (Figure 4b–e and Figure S5), but with significant increases in crystal size and reduction in crystallite number at increased Ti and Ca content (Table 5). Further TEM analysis could elucidate their chemical composition. Apart from the possibility of later epitaxial growth, phase-separated domains rich in displaced Ti, Ca, or other cations are highly likely and have been demonstrated previously at higher Ti content [27]. This may give rise to changes in glass viscosity influencing structural rearrangement and creating boundaries between the different glass species and sites for induced crystallization [34]. Zanotto [35], however, indicated that liquid phase separation (LPS) pushed the glass matrix composition closer to the crystal phase stoichiometry. In the current formulations, LPS may have encouraged nanoscale seeds/crystallites (Na/Ca titanates), encouraging the heterogenous growth of a leucite phase, as many of the observed spherical domains were in association with the leucite crystal or their interfaces (Figure 4a–e and Figure S5). The Tg and activation energy (Table 3) of the experimental glasses increased with increasing TiO_2_/CaO content, which is linked to the compositional and glass structural differences. Numerous models have been suggested to predict and explain glass viscosity, Tg relations in glasses and their effects [36]. Lower activation energies (186.8–201.8 KJ^−1^, Figure 1) were found in the present glasses when compared to previous compositions with lower TiO_2_/CaO ratios (213.3 KJ^−1^) [37]. The activation energies calculated should consider the temperature dependence of the activation energy estimated via the Kissinger method, as well as any potential errors [38].

The nucleation agent or its combination with a divalent cation appears to lower the activation energy of crystallization in these novel glass-ceramics [39]. Reduced activation energy (125 KJ^−1^) was reported for leucite glass-ceramics synthesized (via kalsililite) by a sol-gel process and following a diffusion-controlled nucleation and three-dimensional growth process [40]. Löschmann et al. suggested that an interfacial region lower in viscosity between the glass and the growing crystallite was associated with activation energy/crystal growth changes [41]. DSC runs at different particle sizes for glass-ceramics B1–B2, which indicated a surface crystallization mechanism indicated by the peak shifts in Tp for differing powder sizes (Figures S2 and S3). It is interesting to note that at 3 mol% TiO_2_/CaO addition there was a 1 °C difference in peak position between the 45 and 300 μm powders (1.8 °C difference between 45 and 125 μm), which might indicate a crystallization mechanism change and with a change in unit cell (Table 4) and crystal morphology. A glass containing an effective nucleating agent might be expected to produce only minor differences in DSC peak positions, indicating its susceptibility to a bulk nucleation mechanism [42]. This is in a system which is known to crystalize via a surface crystallization mechanism [13].

Nucleation holds longer than 0.5 h in glass B1 (1 mol% TiO_2_/CaO) increased crystallite number and produced a statistically significant reduction in median crystal size (*p* < 0.05) across groups, and therefore appeared the most efficient in terms of nuclei formation. At higher (1–2 mol%) TiO_2_/CaO (B2–B3) addition there was an increase in median crystallite size and a reduction in number (Table 4). The increased crystal growth temperature and duration may be a factor in the increased crystal growth, allowing the maturation into high aspect ratio fibers and cross-shaped structures (Figure 3d–f, Figure 4c–f and Figure S5). Longer nucleation holding times at higher TiO_2_/CaO (2–3 mol%) addition may have produced more nucleation sites, but Ostwald ripening [43,44], crystal impingement and coalescence would have masked these effects (Figure 3d,f). Interface kinetics influence crystal growth via mechanisms of normal, 2D, screw dislocation growth, or a combination of these processes which were modeled [45]. It was noted that the leucite phase was difficult to model due to the crystals embedded in a droplet zone differing from the original glass composition. This will certainly influence the crystal growth conditions or any heteroepitaxial growth on another phase [42]. Wisniewski and Rüssel [46], nevertheless, indicated the importance of crystal growth kinetics leading to specific orientations, with faster growth (viscous fingering process) via the c–axes tilted by 45 degrees (to main growth direction) or slower growth via the c–axes parallel to the primary growth path. The increased crystallization temperature differences (35–48 °C, Table 2) between B1 and B2–3 glass-ceramics may also have led to changes in crystal growth, developing a more dendritic morphology.

Leucite glass-ceramics with a 1 h nucleation hold were selected for BFS testing due to the combination of the highest crystallite number (Table 4) and smallest median crystallite size. This was in line with studies indicating the optimum nucleation hold would yield glass-ceramics with fine-grained microstructures [47], with advantages of low wear, high mechanical strength and translucency [17]. Mackert et al. [48] suggested a reduction in leucite crystal size (<4 μm) would promote increases in leucite glass-ceramic flexural strength by limiting glass micro-cracking due to tensile stress [49,50]. The glass-ceramics (B1–B3) produced high BFS (211.6–235.1 MPa) in a similar range to glass-ceramics produced using additional ball milling (BFS = 225–255 MPa) to control crystallite size (PS = 0.185–0.048 μm^2^), but with higher reliability for B1 and B3 glass-ceramics (Table 5). This may be associated with the differing crystal morphology, high leucite area fraction (57.4–55.1%) and high TEC values (Table 3). There was also a change in aspect ratio of the unit cell, with a reduction in a–axis and increase in c–axis dimensions throughout the glass-ceramic series (increasing TiO_2_/CaO addition), with increases in dendrite and cross-shaped crystal growth of high aspect ratio. In particular, the mean *c*–axis unit cell dimension (13.7170) for IPS Empress Esthetic [16] was lower than in the experimental groups. According to Table 4, the calculated crystal strains were largely maintained in the experimental glass-ceramics when compared with a previously reported commercial material (Ceramco-3) with a significantly lower BFS [51]. The B3 glass-ceramic had reduced *c*–axis crystal strain compared with the other test groups (Table 4), which may be related to the change in crystal morphology and development of longitudinal cracks and stress relief within the elongated crystals. This did not, however, have a detrimental effect on the BFS (235.1 MPa) or its remarkable reliability (m = 18.9), as the tangential compressive stresses were maintained in the glassy matrix, with no matrix microcracking evident. The high aspect ratio of fibers and cross-shaped features present in most of the glass-ceramics should encourage crack pinning, bowing, bridging and the possibility of crack tip shielding mechanisms [8,10,52]. Further work is needed to explore if the fracture toughness of these materials has been improved.

The absence of matrix microcracking in the experimental glass-ceramics (Figure 3a–f) suggests a favorable thermal expansion match between the leucite crystals and glassy matrix, which contributes to crack deflection and superior mechanical performance [53]. The novel glass-ceramics with added TiO_2_/CaO (1–3 mol%) produced translucent glass-ceramics with a bulk leucite phase, with unique microstructure that can be processed using sintering, heat extrusion or computer-aided design/computer-aided manufacturing processes [30] to produce potentially tough and high-strength aesthetic dental restorations.

## 5. Conclusions

The addition of TiO_2_/CaO (1–3 mol%) to a novel aluminosilicate glass led to the crystallization of leucite glass-ceramics of high aspect ratio, with high BFS and reliability for dental applications.

## 6. Patents

Patents resulting from the work reported in this manuscript are US9856165B2 and EP3013762B1.

## Figures and Tables

**Table 1:** Glass compositions (mol%).

Glass	SiO_2_	Al_2_O_3_	K_2_O	CaO	TiO_2_	Na_2_O	Li_2_O	MgO
B1	69.6	10.2	12.5	1.0	1.0	4.0	1.1	0.5
B2	68.2	10.0	12.3	2.0	2.0	3.9	1.1	0.5
B3	66.8	9.7	12.0	3.0	3.0	3.8	1.1	0.5

**Table 2:** The two-step heat treatment temperatures of the experimental glasses.

Glass	Nucleation Temperature (°C)	Crystal Growth Temperature (°C)
B1	606	868
B2	613	903
B3	618	916

**Table 3:** Dilatometry results for the experimental glass-ceramics and glass.

Material	Predicted TEC (°C)	TEC (×10^−6^/K, 100–400 °C)	Glass Transition Temperature (°C)	Softening Point (°C)
B1 glass	10.32	9.93	576.0	664.4
B2 glass	10.35	10.01	582.7	675.3
B3 glass	10.37	10.06	587.8	675.4
B1 glass-ceramic		20.23	471.7	611.8
B2 glass-ceramic		19.61	486.8	657.1
B3 glass-ceramic		24.87	508.7	658.5

**Table 4:** The mean unit cell dimensions for the experimental glass-ceramics after two-step heat treatments.

Glass- Ceramics	Mean *a*–Axis Unit Cell Dimension Å (SD)	Mean *c*–Axis Unit Cell Dimension Å (SD)	Mean Unit Cell Volume Å (SD)	*ɛ* *_a_*	*ɛ* *_c_*
B1	13.1082 (0.0005)	13.7205 (0.0009)	2357.50 (0.21)	0.33%	−0.25%
B2	13.1016 (0.0005)	13.7248 (0.0008)	2355.89 (0.20)	0.28%	−0.22%
B3	13.1009 (0.0006)	13.7319 (0.0009)	2356.88 (0.22)	0.27%	−0.17%

*ɛ_a_* = crystal strain in the *a*–axis; *ɛ_c_* = crystal strain in the *c*–axis.

**Table 5:** Results of the quantitative image analysis for glass-ceramics B1–3 at different nucleation holds, followed by a 1h growth hold.

**Glass-Ceramic B1**	**Nucleation Hold (h)**	**Leucite Area Fraction (%)**	**Median Crystal Size (μm^2^)**	**Interquartile Range (Q1, Q3) (** **μm^2^)**	**Crystal Number**
	0.5	61.4	0.784 ^a/A^	0.352, 1.655	1367
	1	57.4	0.617 ^b/A^	0.304, 1.223	1787
	2	60.3	0.603 ^b/A^	0.294, 1.283	1687
	3	60.3	0.596 ^b/A^	0.262, 1.301	1789
**Glass-Ceramic B2**	**Nucleation Hold (h)**	**Leucite Area Fraction (%)**	**Median Crystal Size (μm^2^)**	**Interquartile Range (Q1, Q3) (** **μm^2^)**	**Crystal Number**
	0.5	56.9	0.950 ^a/B^	0.426, 2.024	910
	1	56.7	0.806 ^b/B^	0.376, 1.584	1004
	2	60.0	1.083 ^a/B^	0.525, 2.354	745
	3	60.6	1.012 ^a/B^	0.494, 1.920	983
**Glass-Ceramic B3**	**Nucleation Hold (h)**	**Leucite Area Fraction (%)**	**Median Crystal Size (μm^2^)**	**Interquartile Range (Q1, Q3) (** **μm^2^)**	**Crystal Number**
	0.5	59.6	1.080 ^a/B^	0.542, 2.347	735
	1	55.1	1.145 ^a/C^	0.506, 2.267	841
	2	55.8	1.080 ^a/B^	0.467, 2.207	822
	3	60.8	1.308 ^b/C^	0.674, 2.369	732

Different superscript letters (lower case) indicate significant differences between nucleation hold times (0.5, 1, 2, 3 h) within each glass-ceramic group (*p* < 0.05). Different superscript letters (upper case) indicate significant differences for the same nucleation hold time (0.5, 1, 2, 3 h) between glass-ceramic groups (B1, B2, B3) (*p* < 0.05).

**Table 6:** BFS and Weibull analyses results for the glass-ceramics.

Glass-Ceramics	Mean Flexural Strength (MPa)	SD (MPa)	Weibull Modulus (m)	C.I. for m (95%)	σ_0_ (MPa)	C.I. For σ_0_ (95%)	r^2^
B1	225.3 ^ab^	24.7	10.9 ^a^	8.8–13.6	235.6 ^ab^	228.9–242.6	0.976
B2	211.6 ^a^	26.6	9.4 ^ac^	7.6–11.9	222.6 ^a^	215.2–230.1	0.962
B3	235.1 ^b^	14.7	18.9 ^b^	14.9–24.1	241.6 ^b^	237.6–245.6	0.974
IPS Empress Esthetic	165.5 ^c^	30.6	6.3 ^c^	5.0–7.9	177.5 ^c^	168.9–186.6	0.977

σ_0_ = characteristic strength; C.I.= confidence intervals; different superscript letters indicate significant differences between groups (*p*< 0.05).

## Data Availability

The data presented in this study are available on request from the corresponding author.

## Supplementary Materials

The following supporting information can be downloaded at: https://www.mdpi.com/article/10.3390/ma17143422/s1, Figure S1. XRD plots for the experimental glasses; Figure S2. DSC traces of glass of B1 for different particle sizes at 20 °C/min; Figure S3. DSC traces of glass of B2 for different particle sizes at 20 °C/min; Figure S4. DSC traces of glass of B3 for different particle sizes at 20 °C/min; Figure S5. SEM photomicrograph of twinned high aspect ratio leucite crystals (B3 GC) and spherical inclusions; Table S1. Details of reagents used for glass batching; Table S2. Details of the unit cell calculations for B1, B2 and B3 leucite glass-ceramics.
