# Supplementary material for: Effect of TiO2 and CaO Addition on the Crystallization and Flexural Strength of Novel Leucite Glass-Ceramics
Source: Materials (Basel). 2024 Jul 11;17(14):3422. doi: 10.3390/ma17143422 (PMC11278028; doi:10.3390/ma17143422)
Supplement: Supplementary file 1 [file materials-17-03422-s001.zip › materials-3029098-supplementary.pdf]

Effect of TiO_2 and CaO addition on the crystallisation and flexural strength of novel Leucite glass-ceramics.

Jamila Almuhamadi[1], Mustafa H. Almusali[1], Xiaohui Chen[2], Antonios L. Theocharopoulos[3], Hawraa F. Alostath[1], Natalia Karpukhina[1], and Michael J. Cattell[1,*]

1 Centre for Oral Bioengineering, Faculty of Medicine and Dentistry, Queen Mary University of London, Turner Street, London E1 2AD, UK; j.almuhamadi@zu.edu.ly; m.h.e.almusali@qmul.ac.uk; h.alostath@qmul.ac.uk; n.karpukhina@qmul.ac.uk; m.cattell@qmul.ac.uk.
2 Division of Dentistry, School of Medical Sciences, The University of Manchester, Manchester, UK; xiaohui.chen@manchester.ac.uk.
3 Biomedical Sciences Department, Dental Technology Division, University of West Attica, Athens, Greece; antheoch@uniwa.gr.
* Correspondence: m.cattell@qmul.ac.uk

Supporting information

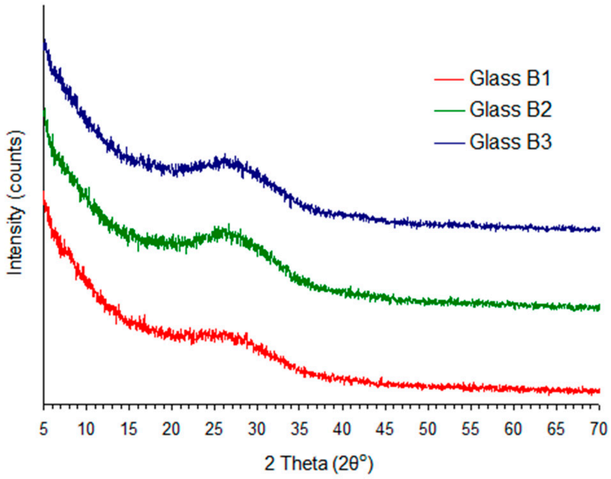

Figure S1. XRD plots for the experimental glasses.

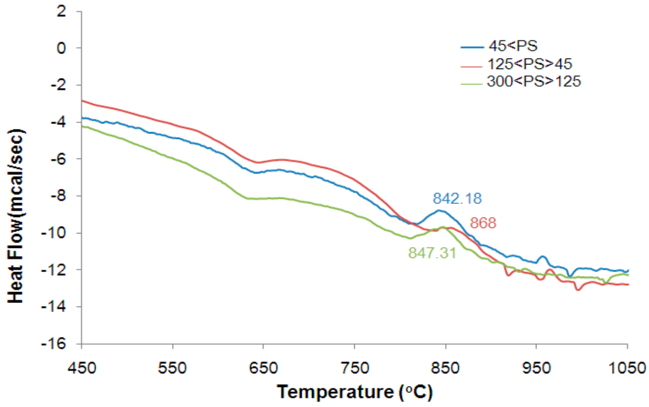

Figure S2. DSC traces of glass of B1 for different particle sizes at 20°C/min.

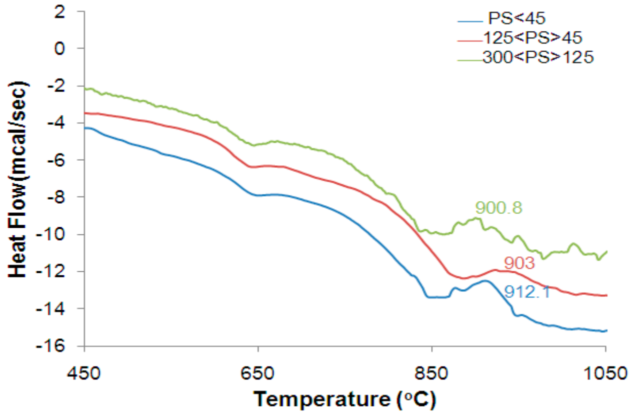

Figure S3: DSC traces of glass of B2 for different particle sizes at 20°C/min.

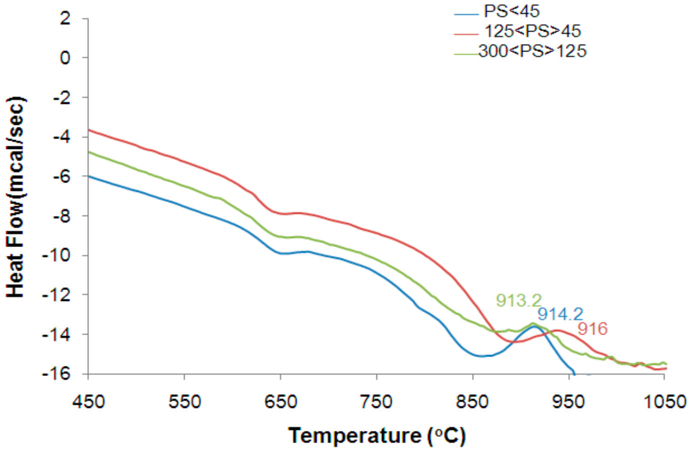

Figure S4: DSC traces of glass of B3 for different particle sizes at 20°C/min.

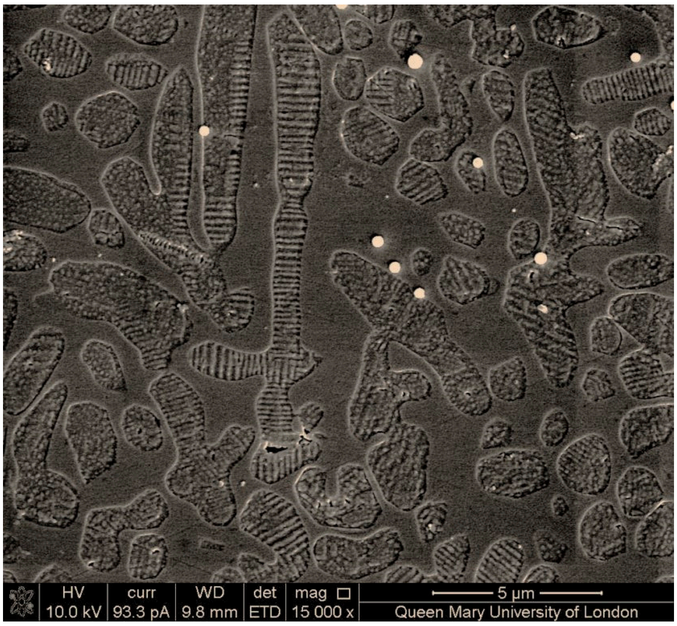

Figure S5: SEM photomicrograph of twinned high aspect ratio leucite crystals (B3 GC) and spherical inclusions.

Table S1. Details of reagents used for glass batching.

Reagent	Description	Manufacturer	Lot no
**Kasil® SS Pwd**	Potassium silicate (SiO_2/K_2 =2.50)	PQ corporation, USA	11-904w -112804
**SS 20 ® Pwd**	Sodium silicate (SiO_2/Na_2O =3.22)	PQ corporation, USA	11-139w -110305
**Aluminium oxide**	Purity: 99.9%	Fluka, Germany	1270233
**Calcium carbonate**	Purity: > = 99.0%	Fluka, Italy	21060
**Lithium carbonate**	Purity: > = 98.0%	Fluka, USA	62372
**Titanium (IV) oxide**	Purity: > = 99.8%	Sigma-Aldrich, USA	232033 (07119ED)
**Magnesium oxide**	Purity: 98.0%	Sigma-Aldrich, USA	220361 (08907BD)
**Silica sand**	Purity: 98.8%	Glassworks service Ltd, UK	

Table S2. Details of the unit cell calculations for B1, B2 and B3 leucite glass-ceramics.

Glass Ceramic	a	b	c	sig(a)	sig(b)	Sig(c)	Volume	sig(V)	a/c	ChiSq	LX	Size/A
B1	13,10816	13,10816	13,72046	0,000524	0,000524	0,000861	2357,503	0,21	0,955373	2,606	14,21	621,1814
B2	13,10161	13,10161	13,72477	0,000509	0,000509	0,000834	2355,886	0,203	0,954596	2,473	13,75	641,9627
B3	13,10094	13,10094	13,73194	0,000546	0,000546	0,000888	2356,875	0,22	0,954048	2,155	14,06	627,8085
